# Dynamic hyperinflation and intrinsic PEEP in ARDS patients: who, when, and how needs more focus?

**DOI:** 10.1186/s13054-019-2713-1

**Published:** 2019-12-23

**Authors:** Heyan Wang, Hangyong He

**Affiliations:** 1 Department of Critical Care Medicine, The Sixth Hospital of Guiyang, Guiyang City, Guizhou Province China; 2 Department of Respiratory and Critical Care Medicine, Beijing Institute of Respiratory Medicine, Beijing Chao-Yang Hospital, Capital Medical University, No. 8 Gongren Tiyuchang Nanlu, Chaoyang District, Beijing, 100020 China

Dear editor,

We read with great interest of the report by Coppola and colleagues [1] about the presence and possible factors of dynamic hyperinflation and intrinsic positive end-expiratory pressure (PEEP) in severe acute respiratory distress syndrome (ARDS) patients. They suggested that in sedated, paralyzed ARDS patients without a known obstructive disease, the amount of intrinsic PEEP during lung-protective ventilation is negligible and does not influence respiratory mechanical properties. However, some details about who, when, and how for monitoring and managing dynamic hyperinflation and intrinsic PEEP in ARDS patients is still needed to be defined.

First, who needs more attention for monitoring dynamic hyperinflation and intrinsic PEEP with ARDS? During lung-protective ventilation in ARDS, when a respiratory rate (RR) up to a maximal of 35 breaths/min was needed to provide a minute ventilation that minimized hypercapnia and respiratory acidosis, the shortening of the expiratory time consequent to the higher RR may generate substantial intrinsic PEEP [2, 3]. In the study by Coppola et al. [1], average RR was only 16 breaths/min. Therefore, severe ARDS patients with a high RR requirement for hypercapnia and respiratory acidosis are at high risk of dynamic hyperinflation and intrinsic PEEP, which needs more investigation.

The second question is when should we focus on dynamic hyperinflation and intrinsic PEEP with ARDS? In the article reported by Coppola et al. [1], only paralyzed patients in the very early stage of ARDS were investigated. However, in deeply sedated ARDS patients without paralysis, the respiratory entrainment with reverse triggering may cause breath stacking in deeply sedated non-paralyzed ARDS patient, which led to volumes and pressures that were incompatible with lung-protective ventilation, and may lead to intrinsic PEEP and dynamic hyperinflation [4].

Finally, how to recognize and calculate intrinsic PEEP in patients with ARDS, especially in patients without neuromuscular blocking agents. In Coppola’s report [1], intrinsic PEEP was defined as the total PEEP minus the external PEEP, and intrinsic PEEP decreased when external PEEP was raised. Expiratory flow limitation and airway closure may be two factors mainly responsible for the development of intrinsic PEEP in ARDS patients, and the response to a raising external PEEP might be due to airway closure and flow limitation at low PEEP and an airway opening pressure at high PEEP. Thus, reliability of the calculation for intrinsic PEEP in Coppola’s study may not be valid under condition of flow limitation, which can occur in patients with ARDS. A measurement of intrinsic PEEP at zero PEEP should be more accurate. Furthermore, for patients with spontaneous breath, an increasing number of reports indicate that measurement of intrinsic PEEP can be obtained both with advanced monitoring systems (esophageal and gastric manometry, diaphragm electromyography, electrical impedance tomography) and, with some limitations, with simple airways occlusion maneuvers in patients with spontaneous breath [5].

Therefore, details about who, when, and how to investigate intrinsic PEEP and dynamic hyperinflation in ARDS patients are still needed to be evaluated. Further researches are needed for early recognition and better measurement of intrinsic PEEP and dynamic hyperinflation in this population.

## Data Availability

Not applicable.
