# Supplementary material for: Dynamic hyperinflation and intrinsic PEEP in ARDS patients: who, when, and how needs more focus?
Source: Crit Care. 2019 Dec 23;23:422. doi: 10.1186/s13054-019-2713-1 (PMC6929493; doi:10.1186/s13054-019-2713-1)
Supplement: Related texts

# Authors reply to: “Dynamic hyperinflation and intrinsic PEEP in ARDS patients: who, when, and how needs more focus”

**Authors:** Coppola Silvia, Caccioppola Alessio, Froio Sara, Ferrari Erica, Gotti Miriam, Formenti Paolo, Chiumello Davide

**Affiliations:** 3 grid.415093.a Department of Anesthesia and Intensive Care, ASST Santi Paolo e Carlo, San Paolo University Hospital, Via Di Rudinì, Milan, Italy; 4 0000 0004 1757 2822 grid.4708.b Department of Health Sciences, University of Milan, Milan, Italy; 5 0000 0004 1757 2822 grid.4708.b Coordinated Research Center on Respiratory Failure, University of Milan, Milan, Italy

We appreciate the time the authors have taken to read our recent article published in critical care on the dynamic hyperinflation and intrinsic positive end-expiratory pressure in ARDS patients [1].

The development of dynamic hyperinflation and intrinsic PEEP is usually generated by the presence of one or by a combination of these variables such as the expiratory flow limitation, the increase in expiratory resistance, the alteration in respiratory mechanics (resistance and compliance) and the ventilator setting (respiratory rate) [3, 6].

Concerning the respiratory rate in promoting the dynamic hyperinflation, we found that the respiratory rate in our population was very similar to that found by Bellani et al. in the “Lung Safe” study (18 vs. 18 bpm, respectively), thus we can assume that our data reflect the common management of ARDS patients [7].

We completely agree that the higher is the respiratory rate, the higher is the possibility to increase dynamic hyperinflation and the intrinsic PEEP [3, 6]. Our patients were sedated and paralyzed, thus there was no double trigger or any form of asynchrony which could have increased the tidal volume or minute ventilation; moreover, the ventilatory setting was maintained fixed throughout the entire study period. We measured the intrinsic PEEP by the occlusion of the airway at the end expiration for a period of 3–5 s, this pressure should represent the average pressure (static intrinsic PEEP) in the different lung regions. Although we did not measure the expiratory flow limitation based on the pressure volume loop, the intrinsic PEEP did not clinically change by increasing PEEP suggesting a minimal role of expiratory flow limitation in the development of intrinsic PEEP [8].
